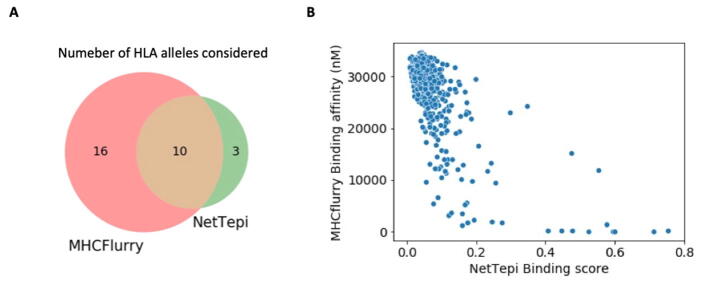# Evidence of a SARS-CoV-2 double Spike mutation D614G/S939F potentially affecting immune response of infected subjects

**DOI:** 10.1016/j.csbj.2022.01.021

**Published:** 2022-01-21

**Authors:** Sara Donzelli, Francesca Spinella, Enea Gino di Domenico, Martina Pontone, Ilaria Cavallo, Giulia Orlandi, Stefania Iannazzo, Giulio Maria Ricciuto, ISG Virology Covid Team, Raul Pellini, Paola Muti, Sabrina Strano, Gennaro Ciliberto, Fabrizio Ensoli, Stefano Zapperi, Caterina A.M. La Porta, Giovanni Blandino, Aldo Morrone, Fulvia Pimpinelli

**Affiliations:** a Oncogenomic and Epigenetic Unit, IRCCS Regina Elena National Cancer Institute, Rome, Italy; b Eurofins Genoma Group Srl, Via di Castel Giubileo, 11, 00138 Rome, Italy; c Department of Microbiology and Virology, IRCCS San Gallicano Dermatological Institute, Rome, Italy; d Department of Prevention, Hygiene and Public Health ASL RM 3, Rome, Italy; e Emergency Department ASL Rome 3, GB Grassi Hospital, Ostia Lido, Rome, Italy; f Department Otolaryngology Head and Neck Surgery, IRCCS Regina Elena National Cancer Institute, Rome, Italy; g Department of Biomedical, Surgical and Dental Sciences, “Università degli Studi di Milano”, Milan, Italy; h SAFU Unit, IRCCS Regina Elena National Cancer Institute, Rome, Italy; i Scientific Direction, IRCCS Regina Elena National Cancer Institute, Rome, Italy; j Center for Complexity and Biosystems, Department of Physics, University of Milan, Via Celoria 16, 20133 Milano, Italy; k CNR – Consiglio Nazionale delle Ricerche, Istituto di Chimica della Materia Condensata e di Tecnologie per l'Energia, via R. Cozzi 53, 20125 Milano, Italy; l Center for Complexity and Biosystems, Department of Environmental Science and Policy, University of Milan, via Celoria 26, 20133 Milano, Italy; m CNR – Consiglio Nazionale delle Ricerche, Istituto di Biofisica, via Celoria 26, 20133 Milano, Italy; n Scientific Direction, IRCCS San Gallicano Dermatological Institute, Rome, Italy

**Keywords:** SARS-CoV-2, Spike mutations, D614G, S939F, Immune response

## Abstract

**Objectives:**

Despite extensive efforts to monitor the diffusion of COVID-19, the actual wave of infection is worldwide characterized by the presence of emerging SARS-CoV-2 variants. The present study aims to describe the presence of yet undiscovered SARS-CoV-2 variants in Italy.

**Methods:**

Next Generation Sequencing was performed on 16 respiratory samples from occasionally employed within the Bangladeshi community present in Ostia and Fiumicino towns. Computational strategy was used to identify all potential epitopes for reference and mutated Spike proteins. A simulation of proteasome activity and the identification of possible cleavage sites along the protein guided to a combined score involving binding affinity, peptide stability and T-cell propensity.

**Results:**

Retrospective sequencing analysis revealed a double Spike D614G/S939F mutation in COVID-19 positive subjects present in Ostia while D614G mutation was evidenced in those based in Fiumicino. Unlike D614G, S939F mutation affects immune response by the slight but significant modulation of T-cell propensity and the selective enrichment of potential binding epitopes for some HLA alleles.

**Conclusion:**

Collectively, our findings mirror further the importance of deep sequencing of SARS-CoV-2 genome as a unique approach to monitor the appearance of specific mutations as for those herein reported for Spike protein. This might have implications on both the type of immune response triggered by the viral infection and the severity of the related illness.

## Graphical abstract

**Figure:**
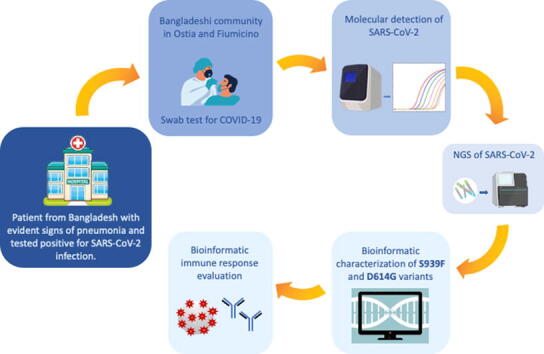

## 1 Introduction

Betacoronaviruses are responsible of the last three major pathogenic zoonotic diseases occurred in the past two decades [1]. Indeed, severe acute respiratory syndrome (SARS-CoV) emerged in 2002 and exhibited 10% mortality of infected people [2]. Middle East respiratory syndrome coronavirus (MERS-CoV) appeared in 2012 with 35% mortality [3]. To date, SARS-CoV-2 records worldwide over than 80 millions of infected people with around 2 millions deaths. Since SARS-CoV-2 pandemic is still active, the related numbers grow daily. Coronavirus entry into host cells is pivotal for viral infectivity and pathogenesis. It also represents of major determinant for immune surveillance and a key target for therapeutic intervention. SARS-CoV-2 enters host cells of high and low respiratory tracts binding ACE2, a cell surface receptor for viral attachment [4]. Subsequently TMPRSS2 internalization protease primes S protein [4]. SARS-CoV S1 contains a Receptor-binding domain (RBD) that specifically binds to hACE2 receptor. RBD status constantly oscillates between standing-up conformation for receptor binding to lying-down position for immune evasion [5]. The crystal structure of the complex between SARS-CoV-2 RDB and h-ACE2 receptor has been recently solved [6]. It revealed subtle differences between previously identified SARS-Co-V RBD and SARS-CoV-2 RBD to recognize hACE2. This leads to the increased binding affinity of SARS-CoV-2 to the receptor and determines its severe effects of the infected cell types. Moreover, compared with other SARS-related coronaviruses (SARSr-CoVs), SARS-CoV-2 possesses a unique furin cleavage site (FCS) in its spike protein that is highly functional and that increases the efficiency of virus infection into cells [7].

Herein we identified by retrospective next-generation sequencing analysis a SARS-CoV-2 double Spike mutation D614G/S939F in 16 respiratory samples from occasionally employers within the Bangladeshi community present in Ostia. SARS-CoV-2 Spike D614G mutation was evidenced in the members of the Bangladeshi community living in the close town Fiumicino who were frequently in contact with those members found positive for Spike D614G/S939F double mutation in Ostia. We also found that unlike D614G, S939F mutation affects immune response through the slight but significant modulation of T-cell propensity and the selective enrichment of potential binding epitopes for some HLA alleles.

## 2 Results

### 2.1 Identification of a SARS-CoV-2 double Spike mutation D614G/S939F

RA, a 44-year old male from Bangladesh was admitted at the Emergency Unit of Grassi Hospital in Ostia-ASL Rome 3 on 7.16.2020 with the following clinical parameters and symptoms: normal ECG, oxygen saturation values of 97%, fever and left flank pain. The patient referred, exhibited evident signs of pneumonia and tested positive for SARS-CoV-2 infection. Since RA lived in Ostia in the same house with 8 members of the Bangladeshi community, the COVID-19 team of ASL-Rome 3 tracked and tested all members of SARS-CoV-2 infection. All roommates tested positive for SARS-CoV-2 infection but unlike RA did not show any evident sign of pneumonia. The molecular detection of SARS-CoV-2 was performed at the Virology and Microbiology Unit of San Gallicano Institute in Rome. For working reasons some of the members of the Bangladeshi group in Ostia interacted frequently with a group of Bangladeshi workers located in Fiumicino, a city of 80,000 habitants located at 25 miles from Rome. All these identified co-workers were assessed for SARS-CoV-2 infection on July 2020 and tested positive. All SARS-CoV-2 positive subjects left Italy during the lockdown period (March to May 2020) and returned in Bangladesh. They came back to Italy at the beginning of June 2020 and were occasionally employed in Ostia and Fiumicino. We do not have any records regarding previously swabs performed in Bangladesh. Both, their return to Italy from Bangladesh soon after the Italian lockdown and their occasional employment at different site in Italy prompted us to sequence their SARS-CoV-2 genome and consequently to monitor the potential presence of previously unidentified viral variants. To this end Next Generation Sequencing analysis was performed on 16 respiratory samples (six NPS and seven BAL) from 16 patients obtaining on average 2.0 × 10^6^ reads per sample (range 0.8–4.2 × 10^6^). Mean value and range of coverage for SARS-CoV-2 genome of reads obtained by NGS for each analyzed sample are represented in Supplementary Table 1.

**Fig. 1:**
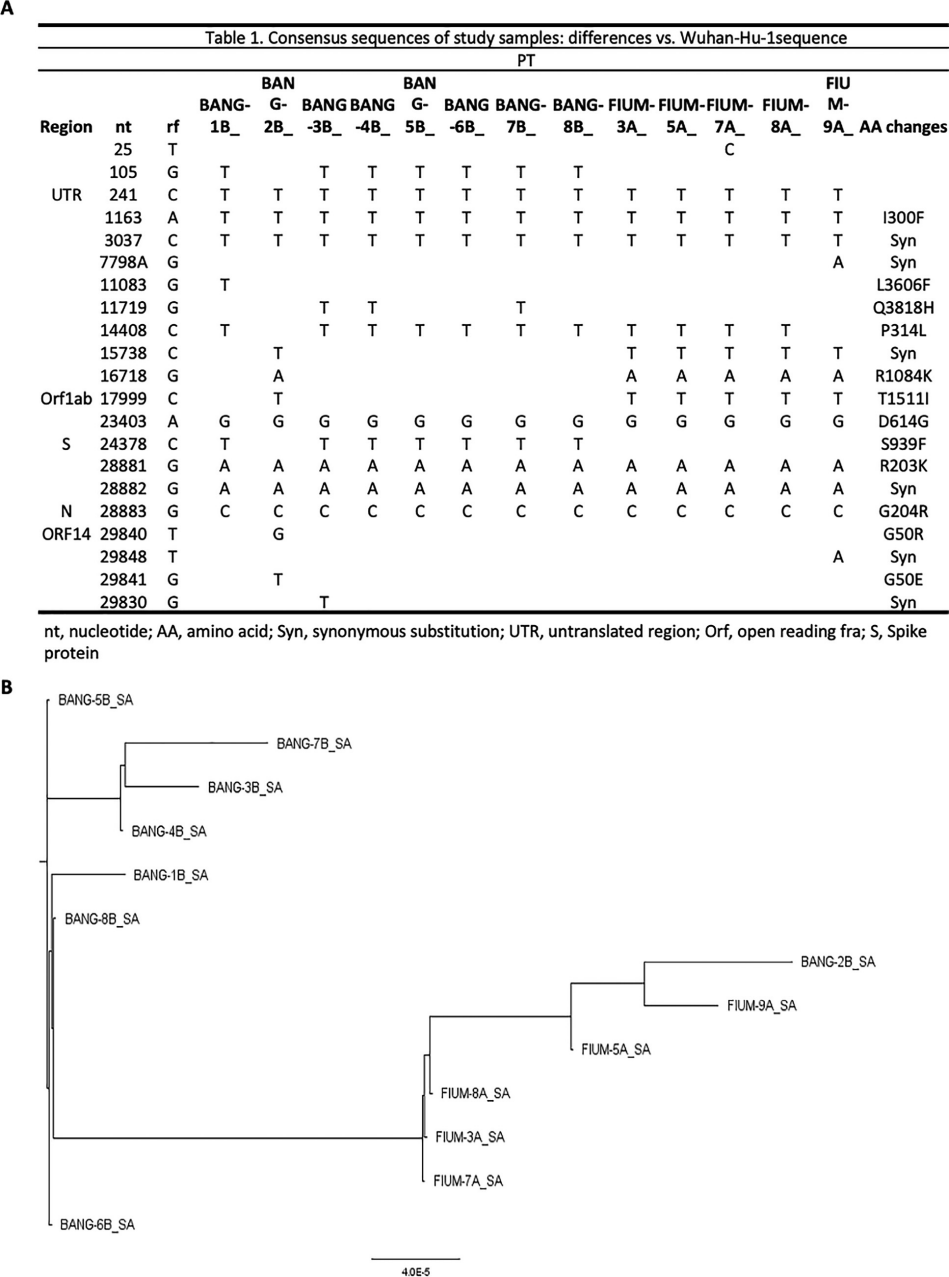
Genetic Variability and phylogenetic analysis of Whole-Genome Consensus Sequences. A. For the mutations analysis, sequences of viral genomes and the reference sequence (GenBank ID NC_045512.2) were aligned with Clustal Omega [31], [32] and analyzed with MEGA X [33]. Nucleotide positions are referred to Wuhan-Hu-1(reference genome MN908947). B. For phylogenetic analysis, we inferred the maximum-likelihood tree using the edge-linked partition model in IQ-TREE (http://www.iqtree.org/) [34], [35].

Consensus sequences are described in the table in Fig. 1A, and differences with the Wuhan-Hu reference genome (GenBank: MN908947) are highlighted. We obtained 13 complete genomes (Fig. 1A). Consensus genomes had a median of 8 substitutions relative to the Wuhan-Hu-1/2019 reference sequence (range 7–10). For phylogenetic analysis, we inferred the maximum-likelihood tree using the edge-linked partition model in IQ-TREE and we identified 2 unique evolutionary lineages in our cohort (lineages was built on the basis of the similarity of the fasta, therefore of the nucleotide sequences, see Methods; Fig. 1B). Most sequenced genomes resemble the lineage B.20 (see methods). We evaluated whether any of the analyzed employees was part of an epidemiologically linked cluster based on illness onset date, positive test status, and work location. We found a correlation between geographic location and mutation set. All employees in the same clusters also had identical or nearly identical consensus genomes, which reflects the low genetic diversity of SARS-CoV-2 at this stage of the pandemic. It is highly unlikely that there are direct transmission pairs in our dataset, but we cannot conclusively rule out coincident transmission linkage. However, the high similarity between one case belong to the group of Bangladeshi (bang 2B) to the group of Fiumicino, suggests that 2B acted as a bridge between the two clusters. All consensus sequences have been submitted to GISAID and GeneBank.

### 2.2 Worldwide geographically distribution of double Spike mutation D614G/S939F

**Fig. 2:**
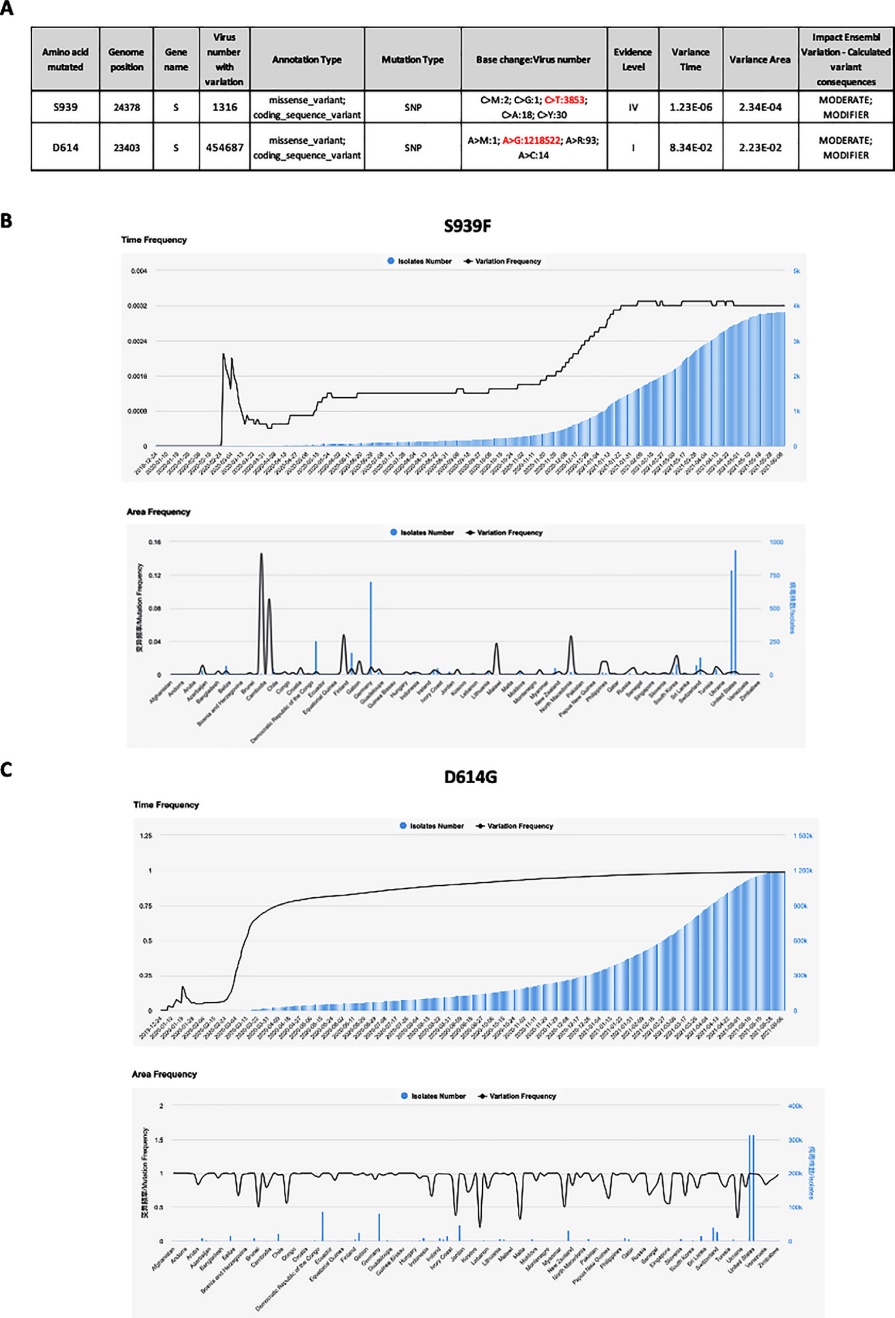
A. S939F and D614G mutations genomic locations and characteristics by 2019nCoVR. The evidence level was graded into I-III according to the number of mutations in high-quality sequences and the density distribution of mutations (population frequency of class I is greater than 0.05, which indicates it is more credible; class II variant sites fall in high-density areas; population frequency of class III is less than 0.05, indicating its low reliability). The Variance Time calculates the population frequency of each mutation site over time, evaluates the variance dispersion of the site by calculating the variance of population frequency at each time point. The Variance Area, calculates the population frequency of each mutation site, evaluates the variance dispersion of the site by calculating the variance of population frequency in each region. The Ensembl Variation - Calculated variant consequences is a prediction of the effects that each allele of the variant may have on each transcript. B-C. Time (upper panel) and Area (lower panel) frequencies of S939F (B) and D614G (C) mutations by 2019nCoVR are indicated. Isolates number is indicated in blue, variation frequency is indicated in black. (For interpretation of the references to colour in this figure legend, the reader is referred to the web version of this article.)

**Fig. 3:**
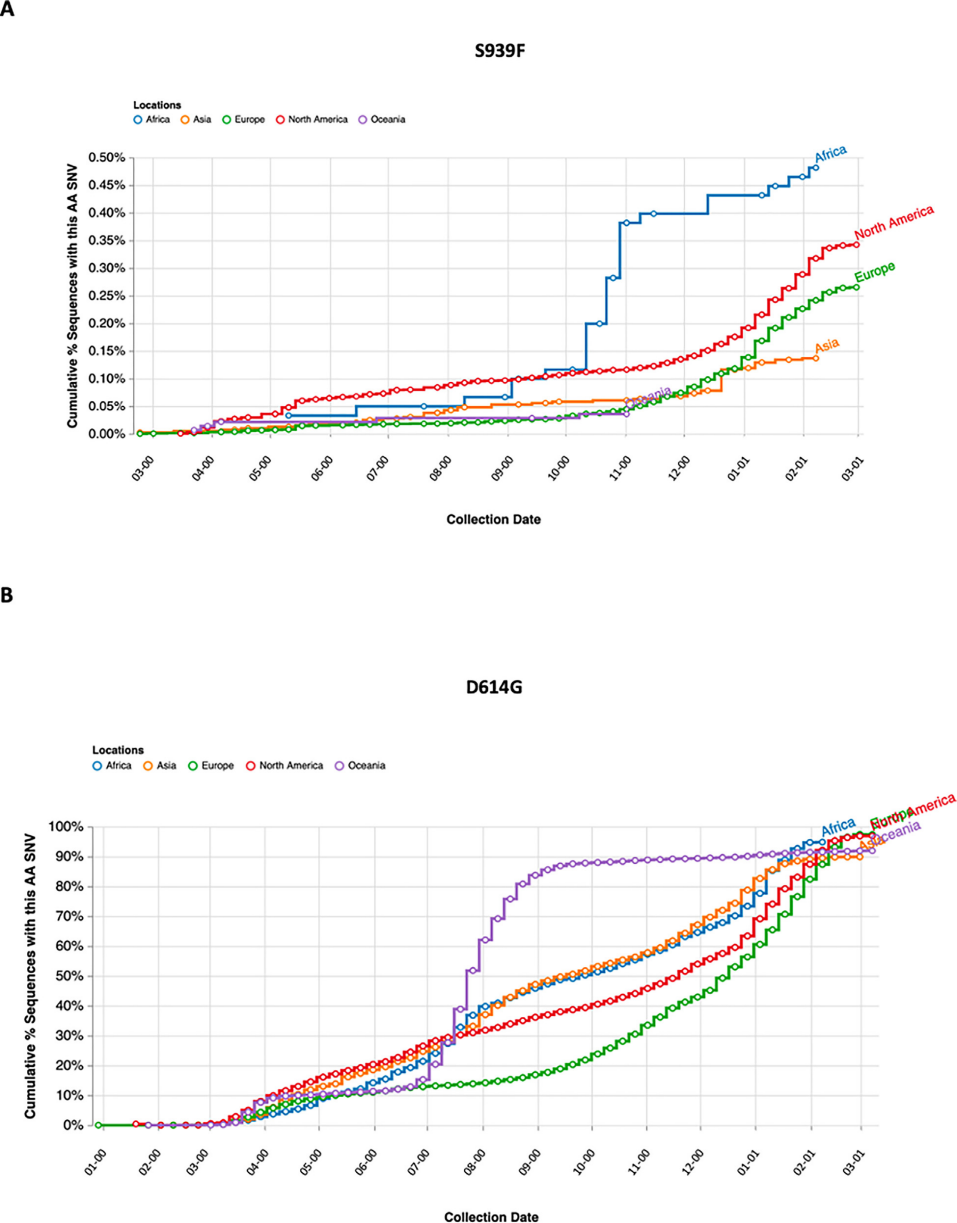
A-B. S939F (A) and D614G (B) mutations occurrence in the five continents of the world from 1 March 2020 to 31 January 2021 by COVID CG.

**Fig. 4:**
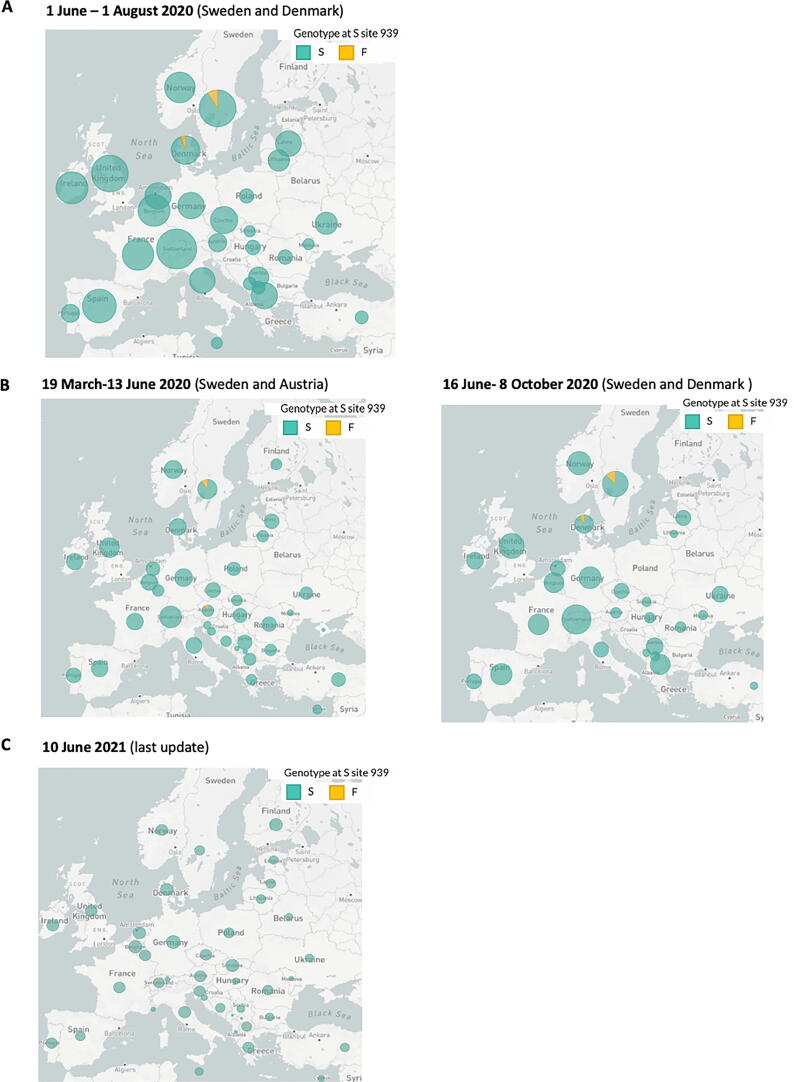
A-C. S939F mutation distribution (indicated in yellow) in Europe at the indicated time intervals by GSAID. (For interpretation of the references to colour in this figure legend, the reader is referred to the web version of this article.)

To further analyze the features of the identified SARS-CoV-2 double mutation, we investigated more in detail each single variant S939F and D614G. By interrogating 2019nCoVR browser we obtained information for both variants, including number of sequences actually deposited, and population frequency. A prediction of the effects that each allele of the variant might have on each transcript was also evidenced (Fig. 2A). In particular, S939F variant, due to nucleotide change C to T in position 24378 of Spike gene, resulted to be about three hundred times less frequent than D614G variant, due to nucleotide change A to G in position 23403 (3853 versus 1218522 counts), determining an evidence level of IV for S939F versus I for D614G. Furthermore, we investigated dynamic patterns of SARS-CoV-2 genomic variants S939F and D614G independently, across different sampling locations over time. As shown in Fig. 2B-C, S939F variant frequency slightly increased over time, reaching 0.0032 at the beginning of June 2021, while D614G variant frequency dramatically increased from 0 at the end of February 2020 to 0.98 at the beginning of June 2021, indicating that this mutated genotype might have higher transmissibility. To figure out where S939F and D614G variants were globally located over time, we interrogated COVID-19 CoV Genetics browser. As shown in Fig. 3A-B both variants resulted to be detected in all continents. Indeed, both are still present in Europe (Fig. 3A-B). Subsequently we assessed S939F variant distribution in Europe. Similar to the analysis performed by Korber and collaborators for the global distribution of D614G variant, we interrogated GISAID to assess S939F variant distribution in Europe [8]. We found that S939F variant was detected in Sweden and Denmark when we evidenced S939F-D614G double mutation in our patient samples, (Fig. 4A). Before (from March to June), it was present in Sweden and Austria (Fig. 4B), while to date it it has been detected only in Switzerland and North Macedonia (Fig. 4C).

Altogether our findings represent the first evidence of a SARS-CoV-2 variant carrying double Spike D614G/S939F in Italy.

### 2.3 Unlike D614G, S939F affects T-cell propensity

**Fig. 5:**
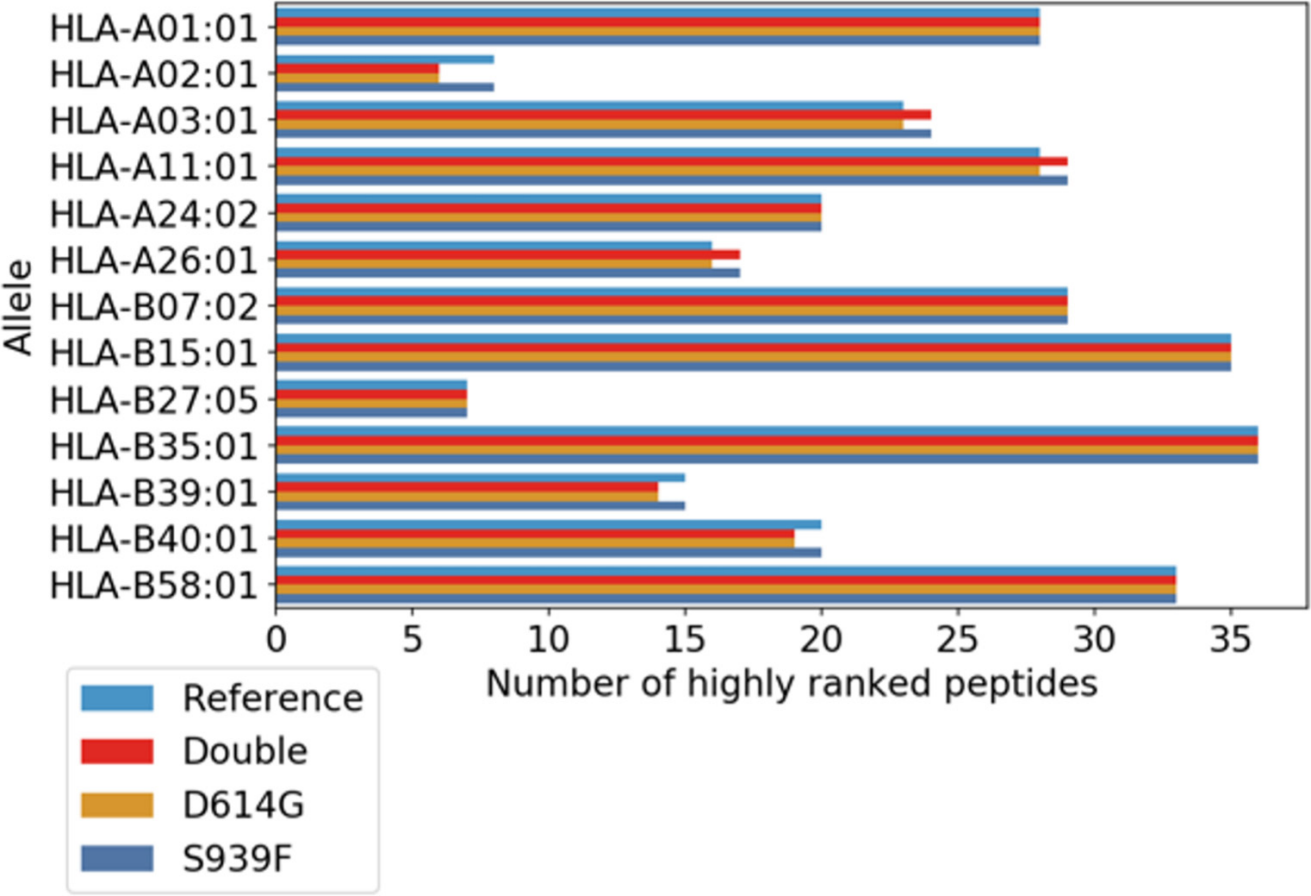
Mutations affect the number of likely T-cell epitopes in a HLA-dependent manner. The figure shows the number of highly ranked peptides from the reference and the mutated (D614G and S939F) SARS-CoV-2 spike protein for a set of HLA alleles, estimated with NetTepi as discussed in the Methods section. For some HLA alleles, the number of highly ranked peptides, the potential T-cell epitopes, differs for the reference and the mutated virus.

The Spike D614G/S939F double mutation is poorly studied and consequently its impact on host infection and patient clinical implications are scarcely known. Here we aim to estimate the effects of the D614G/S939F mutations on the immune response. To this end we adapted to the present experimental aim a computational strategy previously introduced to study the SARS-CoV-2 virus and other similar coronaviruses [9], [10]. In particular, we considered all the potential epitopes associated with the reference and mutated SARS-CoV-2 spike protein. As reported in La Porta & Zapperi 2020 and La Porta & Zapperi 2021, the first step of the process involved a simulation of proteasome activity and the identification of possible cleavage sites along the protein [9], [11], [12]. This resulted in a set of 1549 peptides of length 8–11 for the reference spike protein and 1541 total peptides for both mutations in the protein. We then analyzed the peptides searching for likely epitopes using NetTepi which produced a combined score involving binding affinity, peptide stability and T-cell propensity for 13 supported HLA call I [11]. These three measurements all contribute to the potential that a peptide is a T-cell epitope: binding affinity measures the likelihood that a peptide binds with an HLA, peptide stability measures the ability for the HLA to retain the peptide and T-cell propensity measures whether a peptide is likely to be recognized by a T-cell [11]. The combined score is calculated as a weighted sum of binding affinity, stability and T-cell propensity prediction scores [11]. A high score indicates that the peptide is likely to become a T-cell epitope. From the ranked list of potential epitopes, we selected and counted the highly ranking peptides associated to each HLA allele, as described in the method section. Fig. 5 reports that mutations change the number of potential epitopes for some HLA alleles. In particular, the number of highly ranked peptides is increased by the mutations for HLA-A03:01, HLA-A11:01 and HLA-A26:01, it is decreased for HLA-A02:01, HLA-B39:01 and HLA-B40:01, and it remains unchanged for the other HLA alleles.

**Fig. 6:**
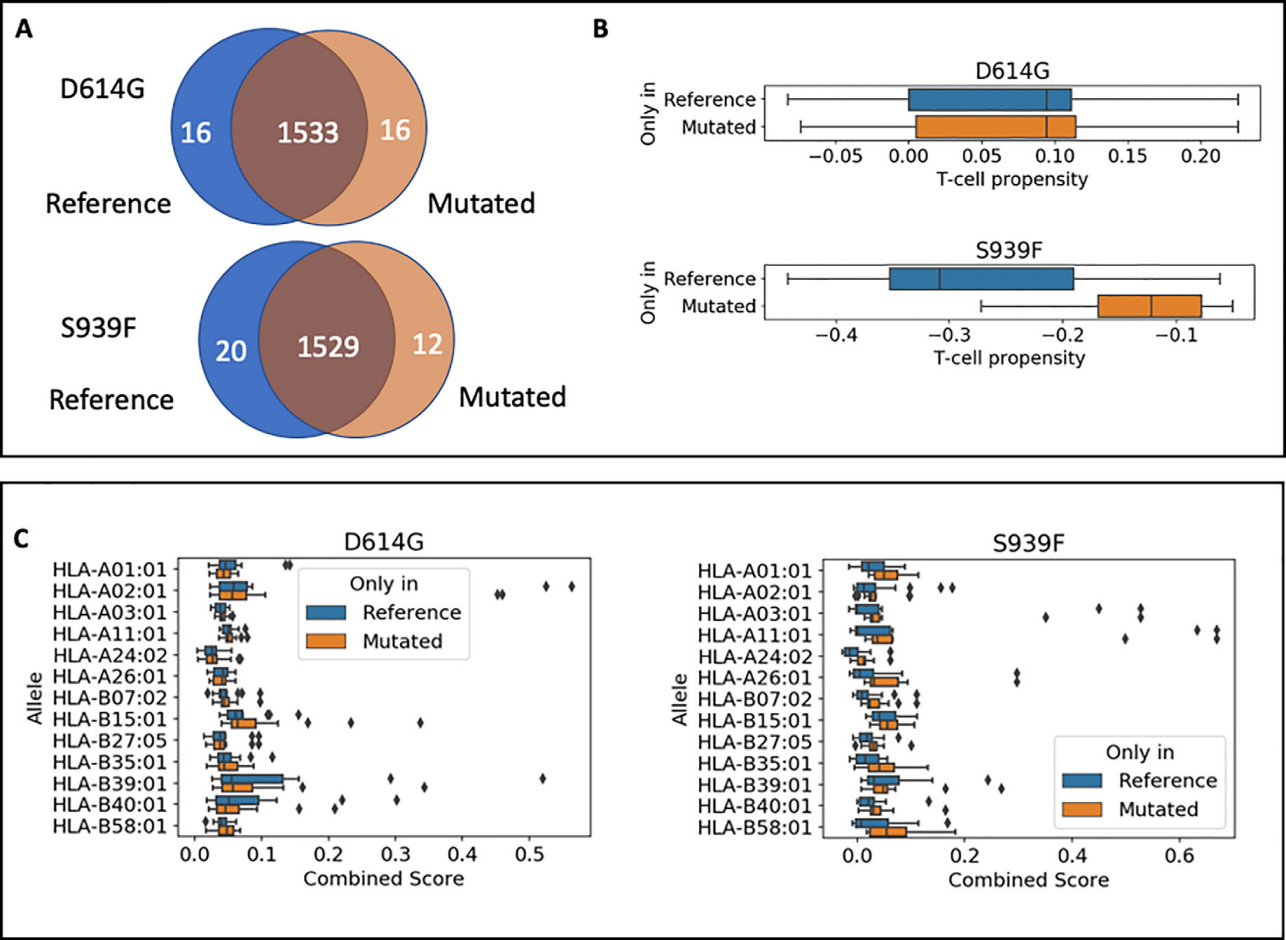
Difference in T-cell propensity and T-cell epitope combined score between reference and mutated peptides. A. After proteasome cleavage simulation, we obtain 1513 peptides that are common between the reference and the mutated virus. A small number of peptides are only present either in the reference virus or in the mutated virus. B. The distribution of T-cell propensities estimated by NetTepi is not affected by the D614G mutation (p = 0.99 according to the Kolmogorov-Smirnov test) while a significant change is observed for the S939F mutation (p = 0.01 according to the Kolmogorov-Smirnov test). The boxplot reports median and quartiles of the data. C. The mutations affect the T-cell epitope combined score of the peptides estimated by NetTepi in a HLA-dependent manner.

The two point mutations D614G and S939F only affected a limited number of peptides, and due to their distance along the sequence no peptide can have more than one mutation. We thus consider the effect of each mutation separately. As shown in Fig. 6A, we can identify a small number of peptides that are either present exclusively in the reference protein (16 for D614G and 20 for S939F) or in the mutated protein (16 for D614G and 12 for S939F) (Table S2). We therefore studied the relevance of these peptides for the immune response. Fig. 6B shows that the T-cell propensity did not change significantly for peptides under the D614G mutation, while the S939F displays a small but significant effect. In particular, the higher T-cell propensity indicates that the mutated spike is more easily recognized by T-cells. In Fig. 6C, we show the combined scores of reference and mutated peptides for the different HLA alleles with some differences observed in an allele dependent manner. In this figure, a decrease in combined score means that the peptide is less likely to be a T-cell epitope.

**Fig. 7:**
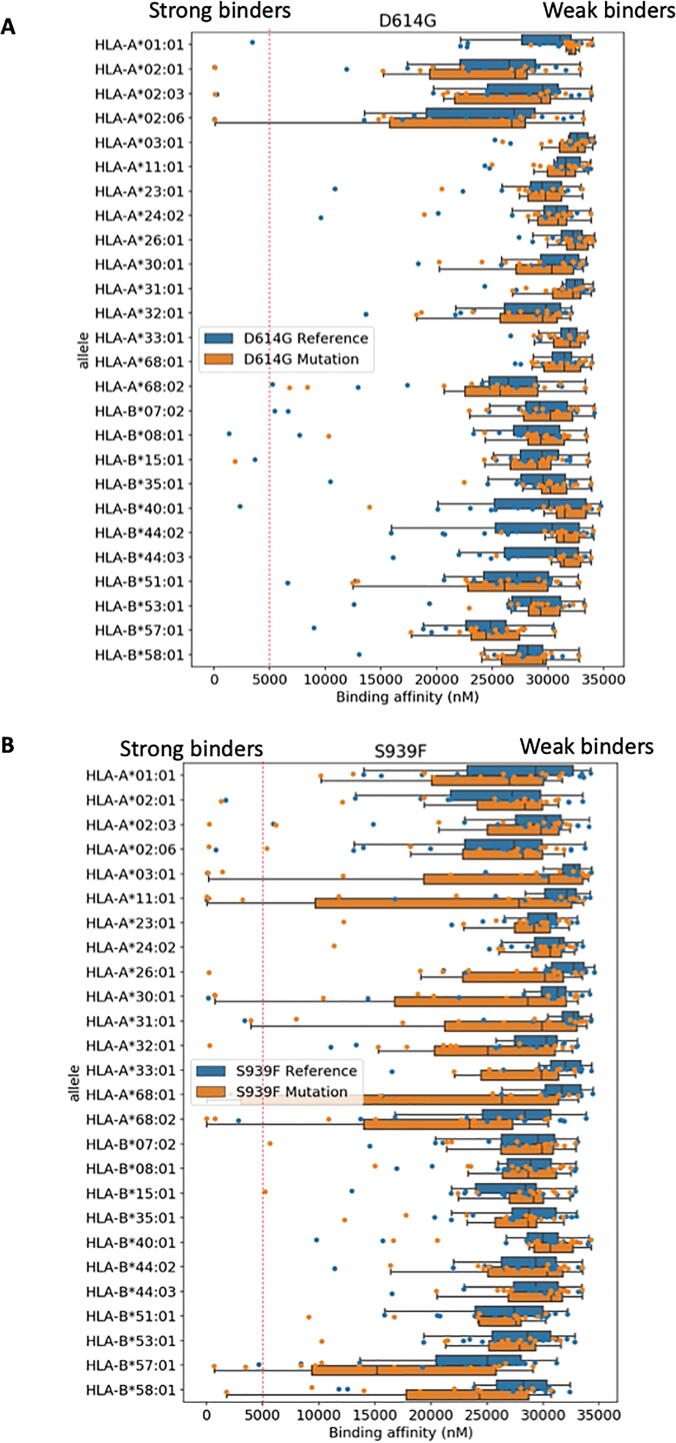
Effect of mutations on binding affinities for a broad range of HLA alleles. We report the binding affinities for the peptides only present in the reference and in the mutated spike protein obtained using MHCflurry 2.0. Individual peptides binding affinities are reported as dots. The boxplot reports median and quartiles of the same data. A. D614G mutation. B. S939F mutation.

Notice that the number of alleles available for NetTepi is rather limited. We report in Table S3 the distribution of HLA-A and HLA-B alleles found in a Bangladeshi population extracted from the allelefrequencies.net website. We can see that NetTepi HLA-A alleles represent 62% of the population and HLA-B only 50%. To obtain a larger coverage of the alleles present in the population we expanded the analysis by considering MHCflurry 2.0 that is able to predict the binding affinity of arbitrary peptides to any HLA molecule using an artificial neural network [13]. We used this tool to compare the binding affinity of the small group of reference and mutated peptides discussed above for 26 HLA class I alleles providing a broad coverage of the human population (see Fig. S1 for a Venn diagram reporting the alleles considered and for a comparison between the predictions of NetTepi and MHCFlurry). In particular, these 26 alleles represent 93% of the Bangladeshi population for HLA-A alleles and 72% for HLA-B alleles. The results reported in Fig. 7A-B show that mutations change the binding landscape only in some cases. For example in the case of the S939F, we could identify some alleles where some new strongly binding peptides emerged in the mutated protein (e.g. HLA-A26:01 or HLA-A32:01), while for the D614G mutation the presence of isolated strongly binding peptides was not affected by the mutation (see HLA-A02:01, HLA-A02:03 and HLA-A02:06).

In aggregate, our findings indicate that Spike mutations may potentially alter CD8 T cell immune response to SARS-CoV-2 thereby affecting the rate of infection and clinical impact.

## 3 Discussion

The widespread diffusion of SARS-CoV-2 depends, at least in part, from its high rate of genome mutation that leads to the appearance of viral variants with different rate of infection and severity of the Covid 19 disease. As for cancer, whose deep deciphering of DNA mutational landscape has been pivotal for the identification of specific driver mutations and for the design of precision medicine therapeutic approaches, the sequencing of the viral genome by using NGS technologies is of pivotal importance.

In the present manuscript, retrospective NGS of SARS-CoV-2 viral genomes revealed a double Spike mutation D614G/S939F in the members of Bangladeshi community located in Ostia as occasional employees. This is the first evidence of the presence of this SARS-CoV-2 double Spike mutation in Italy. Its presence in Europe was previously found in Denmark, Sweden and Croatia. These findings further emphasize the critical need, which is still unmet, to perform massive next generation sequencing of SARS-CoV-2 viral genome to monitor the appearance of novel viral variants and to predict their rate of infection and severity of the related illness in the infected people.

Pre-clinical evidence showed that D614G/S939F double Spike mutation was among those mutations that exhibited higher rate of infection [14]. Multiple studies suggest that T cells are important in the immune response against SARS-CoV-2, and may mediate long-term protection against the virus [15], [16], [17], [18], [19]. Interestingly, we provide novel evidence that the described double Spike mutation affects immune response. To this end, we use computational methods based on artificial neural networks such as NetTepi [11] and MHCFlurry [13]. Indeed, a combined score involving binding affinity, peptide stability and T-cell propensity for 13 supported HLA class I alleles was generated (11). This led to the evaluation of T cell propensity that resulted slight but significantly modulated upon S939F mutation compared to D614G. Furthermore, the binding landscape affinity of predicted

peptides to any HLA molecules was affected by S939F mutation when compared to D614G mutation, that, on the contrary, had no impact on the affinity of strongly binding peptides. One of the most debated issues within the SARS-CoV-2 community is the efficacy of the currently used vaccines against specific viral variants. The generation of tools, as those applied for the identification of a combined score have potential utility as they might also predict viral immune escape of specific SARS-CoV-2 variants. We should also notice that HLA-binding algorithms are widely used in the literature but the results provide only a first indication that particularly in the case of SARS-CoV-2 should eventually be validated experimentally [20]. To date a major question in the SARS-CoV-2 arena relies on the efficacy of the existing vaccines to neutralize emerging viral variants. This emphasizes the need of generating flexible and rapid tools of prediction of immune response upon SARS-CoV-2 infection to instruct not only vaccines but also other antiviral therapeutic approaches.

Collectively, the massive NGS sequencing of viral genomes which leads to the identification of emerging viral variants and the combined evaluation of their impact on the immune response of the infected subject will have a paramount role in fighting both SARS-CoV-2 diffusion and vaccine efficacy.

## 4 Methods

### 4.1 Viral RNA extraction by San Gallicano Institute

RNAs extraction from nasopharyngeal and oropharyngeal swab was performed in two ways. First (to perform routinely Real –Time PCR) by using Bosphore EX-Tract Dry Swab RNA Solution (AnatoliaGeneWork) according to manufacturer’s instructions. Briefly, a dry throat swab from the patient was added to the EX-Tract RNA Solution and vortexed for 60 s. A proportion of this solution was then heated at 95 °C for 8 min. Once cooled this was added directly to the PCR mastermix. Second (to perform NGS), by using the QIAsymphony Virus/Pathogen Kit (QIAGEN), with a final elution of 60ul.

### 4.2 SARS-CoV-2 detection by San Gallicano Institute

For the detection of SARS-CoV-2 in RNAs extracted from nasopharyngeal and oropharyngeal swab we used Bosphore Novel Coronavirus (2019-nCoV) Detection Kit v2 (AnatoliaGeneWork). This kit is a Real-Time PCR-based in vitro diagnostic medical device that allows to detect two regions of the virus in two separate reactions:E gene is used for screening purpose, where 2019-nCoV and also the closelyrelated coronaviruses are detected, and the orf1ab target region is used to discriminate 2019-nCoV specifically. This kit includes also an internal control in order to check RNA extraction, PCR inhibition and application errors.

### 4.3 SARS-CoV-2 detection by genoma laboratory (qualitative analysis)

For the detection of presence/absence of COVID-19, 10 ul of RNA was tested using Allplex™ 2019-nCoV Assay (Seegene) according to manufacturer’s instructions.

The real-time RT-PCR was performed on the CFX96™ (BioRad, California, USA) platform, and subsequently interpreted by Seegene’s Viewer software.

### 4.4 SARS CoV-2 NGS sequencing

Around 5–10 ng of each viral RNA sample was reverse transcribed using SuperScript™ VILO™ cDNA Synthesis Kit (Thermo Fisher Scientific) following the instructions of the Ion Torrent™ Ion AmpliSeq™ Library Kit Plus protocol (Thermo Fisher Scientific). cDNAs have been used for the virus amplification throughout the “Ion AmpliSeq SARS‐CoV‐2 Research Panel” by AmpliSeq™ Technology (Thermo Fisher Scientific). Depending on the number of copies of virus in the extracted samples from 20 to 27 PCR cycles have been performed to get amplicons spanning the virus genome. After the first step of PCR amplification library preparation has been conducted following the Ion Torrent™ Ion AmpliSeq™ Library Kit Plus protocol (Thermo Fisher Scientific). SARS-CoV-2 Ampliseq libraries have been sequenced by using the Ion Chef™ and the Ion Genestudio™ S5 Plus Systems (Thermo Fisher Scientific). Several Ion-supported plug-ins installed in Torrent Suite Software (Thermo Fisher Scientific) have been used for bioinformatic analysis to provide data on coverage sequencing (Table S1), variant calling and annotation, and genome assembly: CoverageAnalysis; VariantCaller, Covid19AnnotateSNPeff, IRMA and AssemblerTrinity [21], [22], [23], [24].

We calculated the mean depth of coverage from the 13 BAM files, at single nucleotide resolution using bed tools. Mapped Reads: number of reads mapped to viral genome; Filtered Reads: percentage of reads failing mapping step; Target Reads: percentage of reads mapped to viral genome; Mean Depth: mean number of time a region has been sequenced; Uniformity: percentage of reads with at least 0.2x of average coverage.

All consensus sequences have been submitted to GISAID with the following accession IDs: EPI_ISL_1181628, EPI_ISL_1257897, EPI_ISL_1224910, EPI_ISL_1257867, EPI_ISL_1257868, EPI_ISL_1257869, EPI_ISL_1257870, EPI_ISL_1257871, EPI_ISL_1257872, EPI_ISL_1257873, EPI_ISL_1257894, EPI_ISL_1257895, EPI_ISL_1257896.

### 4.5 Bioinformatic characterization of S939F and D614G variants

Information about S939F and D614G variants counts and frequency were obtained by 2019nCoVR browser (https://bigd.big.ac.cn/ncov) [25], [26], [27].

Distribution of S939F and D614G variants in the world over time was provided by COVID-19 CoV Genetics browser (https://covidcg.org) [28].

Distribution of S939F variant in Europe at the indicated time points was verified by interrogating GISAID database (https://www.gisaid.org) [29].

### 4.6 Peptide selection by proteasome cleavage.

In the following analysis, we only consider peptides that are likely to be produced by proteasome degradation using NetChop 3.1 [12] a neural network based algorithm that scans proteins for probable cleavage sites of the human proteasome. We perform the scan for the spike protein of the reference virus SARS-CoV-2 and of the mutated virus which includes the two mutations D614G and S939F.

### 4.7 Identification of T cell epitopes

Potential T cell epitopes are identified using NetTepi 1.0 through the server (https://services.healthtech.dtu.dk/service.php?NetTepi-1.0). The method combines estimates for peptide-HLA binding affnity, peptide-HLA stability and T cell propensity [11]. Peptides are then ranked against a set of 200,000 natural peptides to obtain a global rank score. Here we scan all the peptides selected by proteasome simulation with lengths 8–11 from the spike protein of the reference virus SARS-CoV-2 and of the mutated virus, including the two mutations D614G and S939F.

We select highly ranked peptides as those with rank score lower than 2% which are considered “strong binders” (<0.5%) and “weak binders” (<2%). We perform the calculations for all the class I MHC alleles supported by NetTepi, using the default values for the relative weight on stability prediction and the relative weight on T cell propensity prediction.

### 4.8 Prevalence of HLA alleles

The prevalence of HLA alleles in the Bangladeshi population has been identified using Allele frequency net database (AFND) [30].

### 4.9 Estimate of binding affinities

Binding affinities are estimated using MHC flurry 2.0 [13]. We only estimate the binding affinities for peptides selected by proteasome using NetChop 3.1 and that differ between the reference and the mutated (D614G and S939F) SARS-CoV-2 virus.

## CRediT authorship contribution statement

**Sara Donzelli:** Investigation, Writing – review & editing. **Francesca Spinella:** Investigation. **Enea Gino di Domenico:** Investigation. **Martina Pontone:** Investigation. **Ilaria Cavallo:** Investigation. **Giulia Orlandi:** Investigation. **Stefania Iannazzo:** Investigation. **Giulio Maria Ricciuto:** Investigation. **ISG Virology Covid Team:** Investigation. **Raul Pellini:** Investigation. **Paola Muti:** Writing – review & editing. **Sabrina Strano:** Writing – review & editing. **Gennaro Ciliberto:** Supervision. **Fabrizio Ensoli:** Writing – review & editing. **Stefano Zapperi:** Investigation, Writing – review & editing. **Caterina A.M. La Porta:** Investigation, Writing – review & editing. **Giovanni Blandino:** Conceptualization, Writing – review & editing, Supervision, Project administration. **Aldo Morrone:** Supervision. **Fulvia Pimpinelli:** Conceptualization, Writing – review & editing, Supervision, Project administration.

## Declaration of Competing Interest

The authors declare that they have no known competing financial interests or personal relationships that could have appeared to influence the work reported in this paper.

## Figures and Tables

**Supplementary figure 1:**