# Supplementary material for: Evidence of a SARS-CoV-2 double Spike mutation D614G/S939F potentially affecting immune response of infected subjects
Source: Comput Struct Biotechnol J. 2022 Jan 21;20:733–44. doi: 10.1016/j.csbj.2022.01.021 (PMC8780065; doi:10.1016/j.csbj.2022.01.021)
Supplement: Supplementary data 1 [file mmc1.docx]

| **BarcodeName** | **Sample** | **Mapped Reads** | **Filtered Reads** | **Target Reads** | **Mean Depth** | **Uniformity** |
| --- | --- | --- | --- | --- | --- | --- |
| **IonXpress_081** | N39546_COVID | 1112793 | 0.00% | 99.53% | 6980 | 96.46% |
| **IonXpress_082** | N39547_COVID | 234844 | 0.00% | 99.81% | 1457 | 95.79% |
| **IonXpress_083** | N39549_COVID | 987614 | 0.00% | 99.60% | 6222 | 96.45% |
| **IonXpress_084** | N39550_COVID | 249674 | 0.00% | 99.92% | 1530 | 89.33% |
| **IonXpress_085** | N39537_COVID | 260224 | 0.00% | 99.94% | 1558 | 91.99% |
| **IonXpress_086** | N39539_COVID | 1960665 | 0.42% | 99.52% | 12118 | 96.87% |
| **IonXpress_087** | N39541_COVID | 989362 | 0.01% | 99.93% | 6137 | 97.03% |
| **IonXpress_088** | N39542_COVID | 461383 | 0.00% | 99.86% | 2784 | 97.08% |
| **IonXpress_089** | N39544_COVID | 1337375 | 0.00% | 99.92% | 8256 | 95.03% |
| **IonXpress_090** | N39548_COVID | 258789 | 3.49% | 75.42% | 1161 | 93.17% |
| **IonXpress_091** | N39551_COVID | 1899257 | 0.02% | 99.91% | 11755 | 94.12% |
| **IonXpress_093** | N39538_COVID | 321645 | 53.61% | 2.17% | 87 | 34.60% |
| **IonXpress_094** | N39543_COVID | 1441936 | 9.93% | 93.37% | 5685 | 61.98% |
| **IonXpress_095** | N39545_COVID | 1102701 | 8.22% | 85.33% | 4243 | 55.84% |

**Supplementary Table 1**
