# Supplementary material for: Evidence of a SARS-CoV-2 double Spike mutation D614G/S939F potentially affecting immune response of infected subjects
Source: Comput Struct Biotechnol J. 2022 Jan 21;20:733–44. doi: 10.1016/j.csbj.2022.01.021 (PMC8780065; doi:10.1016/j.csbj.2022.01.021)
Supplement: Supplementary data 4 [file mmc4.docx]

**Supplementary Table 1. Mean value and range of coverage for SARS-CoV-2 genome of reads obtained by NGS for each analyzed sample.**

**Supplementary Table 2. List of peptides predicted to significantly alter the immune response.**

**Supplementary Table 3. Prevalence of HLA alleles in the Bangladeshi population**

**Figure S1. Comparison between NetTepi and MHCFlurry.** **A.** Venn diagram describing the number of alleles considered by NetTepi and MHCFlurry. **B.** Cross correlation plot between the binding score of NetTepi and the binding affinity prediction of MHCFlurry.
